# RRApp, a robust randomization app, for clinical and translational research

**DOI:** 10.1017/cts.2017.310

**Published:** 2018-02-19

**Authors:** Chengcheng Tu, Emma K. T. Benn

**Affiliations:** 1 Department of Population Health Science and Policy, Center for Biostatistics, Icahn School of Medicine at Mount Sinai, New York, NY, USA; 2 New York College of Podiatric Medicine, New York, NY, USA; 3 Institute for Translational Sciences (ConduITS), Icahn School of Medicine at Mount Sinai, New York, NY, USA

**Keywords:** Clinical trials, reproducibility, biostatistics, randomization, RCT

## Abstract

While junior clinical researchers at academic medical institutions across the US often desire to be actively engaged in randomized-clinical trials, they often lack adequate resources and research capacity to design and implement them. This insufficiency hinders their ability to generate a rigorous randomization scheme to minimize selection bias and yield comparable groups. Moreover, there are limited online user-friendly randomization tools. Thus, we developed a free robust randomization app (RRApp). RRApp incorporates 6 major randomization techniques: simple randomization, stratified randomization, block randomization, permuted block randomization, stratified block randomization, and stratified permuted block randomization. The design phase has been completed, including robust server scripts and a straightforward user-interface using the “shiny” package in R. Randomization schemes generated in RRApp can be input directly into the Research Electronic Data Capture (REDCap) system. RRApp has been evaluated by biostatisticians and junior clinical faculty at the Icahn School of Medicine at Mount Sinai. Constructive feedback regarding the quality and functionality of RRApp was also provided by attendees of the 2016 Association for Clinical and Translational Statisticians Annual Meeting. RRApp aims to educate early stage clinical trialists about the importance of randomization, while simultaneously assisting them, in a user-friendly fashion, to generate reproducible randomization schemes.

## Introduction

Randomization, as a basic principle of experimental design, plays an essential role in clinical trials [1]. The process of randomization has evolved substantially from randomly allocating patients to treatment arms using a simple toss of a coin [1] to using a comprehensive randomization scheme generated via computer programming. The goal of randomization is to, theoretically, eliminate selection bias in the design and implementation phases of a study to ensure that the observed difference in outcomes between 2 or more treatment groups is due to the treatment/intervention alone [2]. Thus, randomization plays an integral role in reducing threats to internal validity of trial findings when evaluating the efficacy of a treatment or intervention.

Junior clinical faculty and fellows at academic medical institutions across the United States often desire to be actively engaged in and conduct randomized-clinical trials, however these faculty often lack adequate resources and research capacity to appropriately design and implement these trials [3–5]. More specifically, an insufficient background in the design of clinical trials hinders their ability to effectively generate a rigorous randomization scheme to minimize selection bias and yield comparable groups.

We recently conducted an online search, via the Google search engine and PubMed, to evaluate free, online randomization scheme generators. Using the keywords “free best randomization tool” in a Google search on April 7, 2017, we examined the top 50 visited websites, of which we identified a total of 5 randomization scheme generators: randomization.com, randomizer.org, sealedenvelope.com, random.org, and graphpad.com. Yet, we identified several limitations of the web randomization platforms including: a limited number of choices for type of randomization scheme, non-reproducible randomization schemes, complicated user interfaces (UI), and limited free access. Upon searching PubMed using the Mesh Terms “randomization tool” and “computer,” we identified 75 full-text articles published within the past 10 years, among which 3 articles specifically described online randomization scheme generators for clinical researchers: (1) Randoweb, (2) OpenClinica, and (3) MinimRan [6–8]. Among these 3 results, only MinimRan, has limited free access (ie, for the first 10 days) to the public online. However, MinimRan implements covariate-adaptive biased-coin randomization, which has a challenging system to enter massive information regarding the study design and variables of interest, not to mention complicated statistical instructions for nonstatistical users, like junior clinical investigators.

Thus, we developed a free robust randomization app (RRApp) to overcome the aforementioned barriers and provide junior clinical faculty with a convenient tool to generate a rigorous randomization scheme for their clinical trials. The most updated version of RRApp will be freely accessible to the public by December 1, 2017 at https://clinicalresearch-apps.com along with other applications developed by the Center for Biostatistics at the Icahn School of Medicine at Mount Sinai (ISMMS) aimed at increasing the methodological rigor of clinical and translational research. Our objectives for RRApp were 3-fold:

1. To educate users about the importance of randomization in RCTs;
2. To allow users to select and use a variety of commonly used randomization methods;
3. To implement an effective strategy for quality improvement.

### RRApp Randomization Techniques

RRApp currently generates schemes for 6 major randomization techniques: simple randomization, simple stratified randomization, block randomization, stratified block randomization, permuted block randomization, and stratified permuted block randomization. Simple randomization is used to assign patients to treatment groups under a preset probability, without predicting treatment assignments in advance [9]. The commonly used example is tossing a fair coin with an equal probability of heads and tails. This technique is included in most online randomization tools. It is straightforward, easy to understand and implement, and relatively unpredictable. However, the disadvantage is the potential imbalance that can arise during the recruitment process. More specifically, if the study stops recruiting abruptly before meeting the predetermined total sample size, the sample sizes in each treatment arm could be unequal.

Simple randomization is not always optimal when there are known, potentially confounding, prognostic factors such as age group, sex, disease severity, etc. In these cases, simple stratified randomization is highly recommended, which would entail generating a list of treatment allocations for each combination of prognostic factors, also known as strata [9]. For example, imagine we have a hypothetical investigator who is interested in the impact of a specific treatment, as compared to usual care, on lung cancer, but she thinks that the treatment effect might be confounded by sex (ie, male/female) and race (ie, white/nonwhite). The researcher would most likely want to stratify on these factors in the recruitment phase of the study using simple stratified randomization, which would result in a total of 4 treatment allocation subgroups for white males, white females, nonwhite males, and nonwhite females.

Simple stratified randomization can certainly be an optimal design-related consideration for some clinical trials, as in the case of our hypothetical researcher, since it guarantees balance within subgroups and is useful for planned interim analyses [10]. However, excessive strata can lead to extremely small subgroups, especially when the study involves uncommon prognostic factors [9]. In addition, it faces a similar challenge to that of simple randomization, potential imbalance (ie, unequal sample sizes) if the recruitment phase is halted before reaching the predetermined sample size. Block randomization, however, provides a solution to this problem.

In brief, block randomization balances study subjects in small increments, referred to as blocks, to ensure an equal number of subjects in each treatment group over the entire duration of the recruitment phase. For example, in a trial with a total of 2 treatment arms (eg, placebo and treatment), we can denote the total sample size and number of subjects per block as N and b, respectively. Thus, there would be N/b blocks in total, within which b/2 subjects should be allocated to each treatment. This would result in an equal number of patients, N/2, in each treatment group at the end of the recruitment process. The size of the block should be determined by the researcher or collaborating biostatistician a priori and should be a multiple of the number of treatment groups [11]. Therefore, in our example consisting of 2 treatment arms, the block size must be a multiple of 2, like 4, 6, or 8. While block randomization is more complex than the aforementioned techniques, it can be quite advantageous especially when the total sample size is small. It is also suggested that the block size should be large enough to generate sufficient combinations, but not too large to defeat the overarching goal of ensuring an equal number of subjects in each treatment group over time. While beyond the scope of our discussion, it is important to note that researchers should pay close attention to certain covariates, like comorbid conditions, when using block randomization since these factors could compromise the process and cause noncomparable groups [12].

In theory, a relatively small fixed block size could reveal itself accidentally or be figured out by study personnel, which would unfortunately result in “predictable” treatment assignments. Thus, permuted block randomization, consisting of randomizing the block size, is commonly implemented to ensure allocation concealment [9]. For example, in a 2 treatment trial, 2 different block sizes of 4 and 6 could be utilized at random (ie, permutation) over the time of enrollment. This would decrease the chance that a given subject’s treatment assignment could be figured out. However, the same aforementioned block size-related restrictions for block randomization still apply to permuted block randomization.

In some trials, researchers prefer to combine 2 randomization techniques according to their study design such as stratified block randomization (ie, a combination of simple stratified with block randomization), or stratified permuted block randomization (ie, a combination of simple stratified with permuted block randomization). These combined techniques are advantageous since researchers can simultaneously take prognostic factors into consideration while ensuring a balanced design throughout the recruitment process.

## Materials and Methods

### Materials (Programming Software)

RRApp was developed in R 3.3.2 [13], using *shiny* [14], a particularly useful package since it gives R programmers the opportunity to build interactive web applications, without requiring extensive knowledge of other programming languages. Other R packages used for the development of RRApp included *rJava* to make the UI more easily navigable and several packages (ie, *xlsx*, *xlsxjars*, *XLConnect*, *XLConnectJars*) to arrange the randomization scheme output from RRApp in a comprehensive fashion that could be directly imported into Research Electronic Data Capture (REDCap), a secure web application for building and managing online surveys and databases [15–20].

### RRApp Randomization Scheme Input Elements

There are 3 basic input elements required for all of the RRApp randomization techniques: (1) a seed, or numerical pin, to ensure that the randomization schemes generated in RRApp are reproducible, (2) the number of treatment arms, and (3) the sample size. Additionally, when RRApp users choose a randomization technique other than simple randomization or permuted block randomization, they will additionally need to input the block size and/or number of strata. A detailed diagram of the necessary input elements for each randomization technique is provided in Table 1.

**Table 1:** Required input elements for each robust randomization app randomization technique

	Basic elements			Advance elements	
	Seed	Number of treatment arms	Number of people in study	Block size	Number of strata
Simple randomization	✓	✓	✓		
Block randomization	✓	✓	✓	✓	
Simple stratified randomization	✓	✓	✓		✓
Stratified block randomization	✓	✓	✓	✓	✓
Permuted block randomization	✓	✓	✓		
Stratified permuted block randomization	✓	✓	✓		✓

### RRApp UI

**Fig. 1:**
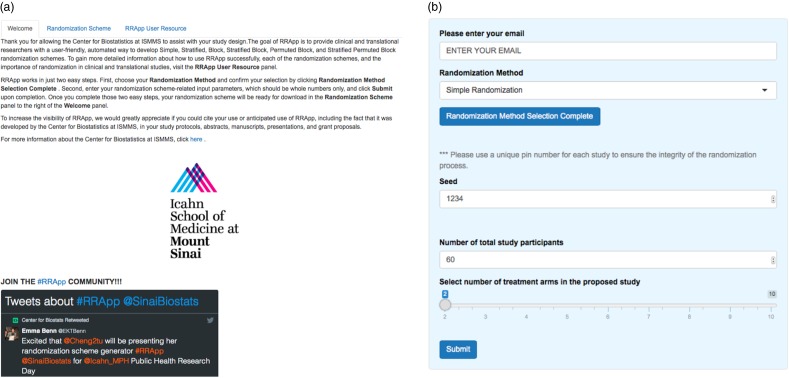
(a) Main panel of user interface for robust randomization app (RRApp) and (b) RRApp side panel for data entry.

Our RRApp UI generally consists of 2 major panels, a main panel and a side panel. The main panel consists of 3 tabs: a “Welcome” tab, a “Randomization Scheme” tab, and a “RRApp User Resource” tab (Fig. 1*a*). The Welcome tab introduces RRApp to users and offers extensive instructions regarding the usage of the side panel. In addition, we incorporated a Twitter feature into the Welcome tab to introduce new users to the larger RRApp community online. The Randomization Scheme tab mainly functions as a download trigger for randomization schemes output from RRApp. Finally, the objectives for the RRApp User Resource tab are 3-fold: (1) to educate RRApp users about the importance of randomization in clinical trials via selected peer-reviewed publications; (2) to offer suggestions about how to choose an appropriate randomization scheme via YouTube videos; and (3) to provide an easy-to-follow user guide of RRApp via a Prezi [21] presentation that can be accessed online (http://prezi.com/vx3pdj5qigbq/?utm_campaign=share&utm_medium=copy&rc=ex0share).

**Fig. 2:**
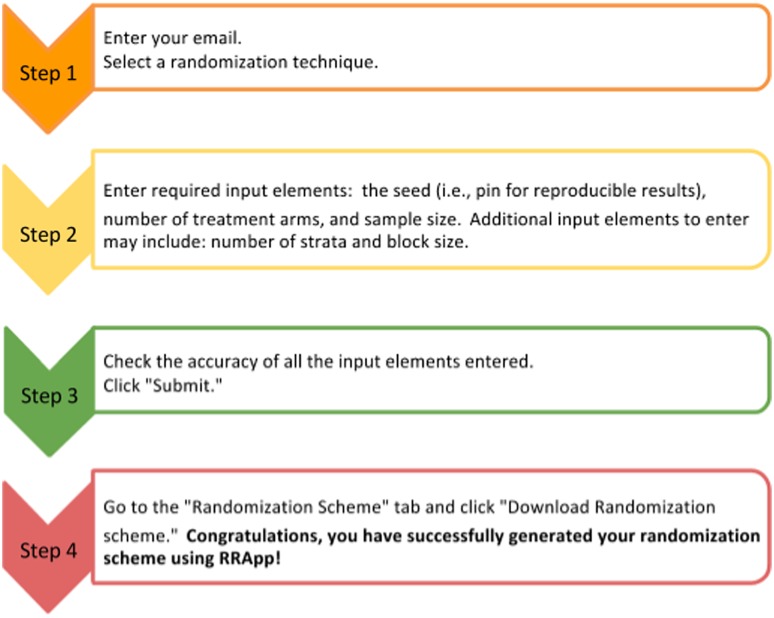
Four steps to generate a randomization scheme in robust randomization app (RRApp).

The side panel (Fig. 1*b*) mainly serves as a data entry portal for the input elements necessary to output the user’s customized randomization scheme using RRApp. There are 4 steps to generate a customized randomization scheme (see Fig. 2). First, users must choose their preferred randomization technique. RRApp users are subsequently asked to enter their email, however submitting this information is optional. The email feature assists us with quality control, since capturing this information will allow us to survey randomly selected users annually to get their constructive feedback about the usability and functionality of RRApp, along with their suggestions about improvements they would like to see us incorporate into the app in the future. Second, conditional on the selected randomization technique, RRApp users are asked to enter the aforementioned input elements. Next, users are asked to thoroughly review the information they have input and then click “submit.” RRApp automatically generates requested downloadable randomization scheme under the Randomization Scheme tab in the main panel. The output randomization scheme is stored as an excel file with a filename that takes into account the date and time at which the randomization scheme was generated.

## Results

RRApp was thoroughly evaluated by faculty and Master-level biostatisticians from the Center for Biostatistics, and early stage clinical and translational investigators in the Department of Population Health Science and Policy at ISMMS. The initial version of RRApp was also presented at the 2016 Annual Meeting for the Association for Clinical and Translational Statisticians (ACTStat). A total of 6 major modifications were suggested for improving the quality and functionality of RRApp in response to the constructive feedback we received from the biostatisticians and clinical investigators at ISMMS, along with the comments made by ACTStat attendees, inclusive of biostatistics faculty members from academic medical centers nationwide. Five of the 6 modifications were incorporated into the final version of RRApp (see Table 2).

**Table 2:** Current status of 6 major modifications to improve the quality and functionality of robust randomization app

	Major changes	Completion status
1	Added an optional user-email feature in the side panel *Aim: To assess user experience and to identify areas for quality improvement via emailing survey at random (from ISMMS)*	Modification complete
2	Provided a REDCap version in the same file *Aim: To apply to a widely used electronic record system (REDCap) in clinical research (from ISMMS)*	Modification complete
3	Incorporate six 3-4 minutes videos regarding each randomization methods *Aim: To educate researchers each randomization technique and its application (from ISMMS)*	In progress
4	Delimited the size of the block to be any multiples of the treatment arms *Aim: To offer more choices for block randomization and stratified block randomization (from 2016 ACTStat meeting)*	Modification complete
5	Notified users of this app should not be involved in the screening process of any studies *Aim: To specify the user requirement to mitigate bias (from 2016 ACTStat meeting)*	Modification complete
6	Incorporated a table of all input elements with the generated randomization schemes *Aim: To serve as a reference (from 2016 ACTStat meeting)*	Modification complete

ISMMS, Icahn School of Medicine at Mount Sinai; REDCap, Research Electronic Data Capture; ACTStat, the Association for Clinical and Translational Statisticians.

Two of the 5 completed modifications, which included collecting the email of users for quality improvement and outputting a randomization scheme that could be directly incorporated into REDCap, were mentioned earlier. The third completed modification consisted of delimiting the block size for users interested in generating a block randomization scheme. Initially, we had more restrictions on the block size users could select from, but now allow users to choose any block size as long as it is a multiple of the number of treatment arms. Our fourth completed modification was to add a warning feature to prevent members of research teams who are actively involved in the screening of patients from using RRApp. Finally, we incorporated documentation of all input elements with the generated randomization schemes into the downloadable file output for the user, so that s/he could refer to these elements and/or reproduce the exact same randomization scheme in the future, if needed. We are currently in the process of adding an additional resource to RRApp which would consists of a series of short, 3-4 minute, instructional videos to ensure that users feel comfortable entering all necessary input elements for each of the randomization schemes available in RRApp. Junior clinical faculty at ISMMS felt that videos of this sort would be very advantageous for them and could help prevent any remaining confusion they might have about the app.

## Discussion

RRApp was successfully developed to provide junior clinical investigators in academic medicine with a free, user friendly, online resource to generate randomization schemes for their clinical trials. It additionally serves as an educational resource for early stage investigators to learn about the importance of randomization in clinical trials and directs users to useful resources to help them easily navigate the app and also determine which of the 6 randomization techniques available through RRApp would be most appropriate for their studies.

There are several strengths of RRApp. First, it overcomes major barriers of other free, publicly-available randomization resources in that it yields reproducible results, has an easy to navigate UI, and an abundance of randomization techniques from which to choose. Second, the fact that RRApp outputs randomization schemes that can be directly imported into REDCap is especially advantageous since REDCap is the standard data capture and data management system used by clinical researchers at NCATS-funded Clinical and Translational Science Award hubs. Third, unlike other online, randomization scheme generators, RRApp gives early stage investigators access to numerous educational resources, so that they can make informed decisions about the most suitable technique to use in order to minimize selection bias in the clinical trials they are conducting. Fourth, since the sustainability of RRApp is highly dependent on whether it is meeting the needs the targeted population of users, the user-informed quality improvements that we make annually will be integral in ensuring the successful continuity and increased ingenuity of the app. A major limitation of RRApp is that it does not include options for dynamic randomization techniques. The generation of these more complex randomization schemes, in our opinion, is not something that should be automated, but should instead result from extensive consultations between the clinical investigators and collaborating biostatisticians.

In summary, RRApp is a unique, publicly-available resource that serves to educate junior clinical faculty in academic medical centers about the importance of randomization, while providing them with a user-friendly platform to generate rigorous and reproducible randomization schemes. While RRApp was initially designed for junior clinical faculty, it is not exclusive to early stage investigators, nor is it exclusive to nonstatistical faculty. RRApp certainly has the potential to serve the larger community of clinical and translational researchers conducting clinical trials both within and outside of academic medical centers, regardless of rank and methodological expertise.
